# Expert consensus on multidisciplinary therapy of colorectal cancer with lung metastases (2019 edition)

**DOI:** 10.1186/s13045-019-0702-0

**Published:** 2019-02-14

**Authors:** Jian Li, Ying Yuan, Fan Yang, Yi Wang, Xu Zhu, Zhenghang Wang, Shu Zheng, Desen Wan, Jie He, Jianping Wang, Yi Ba, Chunmei Bai, Li Bai, Wei Bai, Feng Bi, Kaican Cai, Muyan Cai, Sanjun Cai, Gong Chen, Keneng Chen, Lin Chen, Pengju Chen, Pan Chi, Guanghai Dai, Yanhong Deng, Kefeng Ding, Qingxia Fan, Weijia Fang, Xuedong Fang, Fengyi Feng, Chuangang Fu, Qihan Fu, Yanhong Gu, Yulong He, Baoqing Jia, Kewei Jiang, Maode Lai, Ping Lan, Enxiao Li, Dechuan Li, Jin Li, Leping Li, Ming Li, Shaolei Li, Yexiong Li, Yongheng Li, Zhongwu Li, Xiaobo Liang, Zhiyong Liang, Feng Lin, Guole Lin, Hongjun Liu, Jianzhong Liu, Tianshu Liu, Yunpeng Liu, Hongming Pan, Zhizhong Pan, Haiping Pei, Meng Qiu, Xiujuan Qu, Li Ren, Zhanlong Shen, Weiqi Sheng, Chun Song, Lijie Song, Jianguo Sun, Lingyu Sun, Yingshi Sun, Yuan Tang, Min Tao, Chang Wang, Haijiang Wang, Jun Wang, Shubin Wang, Xicheng Wang, Xishan Wang, Ziqiang Wang, Aiwen Wu, Nan Wu, Lijian Xia, Yi Xiao, Baocai Xing, Bin Xiong, Jianmin Xu, Jianming Xu, Nong Xu, Ruihua Xu, Zhongfa Xu, Yue Yang, Hongwei Yao, Yingjiang Ye, Yonghua Yu, Yueming Yu, Jinbo Yue, Jingdong Zhang, Jun Zhang, Suzhan Zhang, Wei Zhang, Yanqiao Zhang, Zhen Zhang, Zhongtao Zhang, Lin Zhao, Ren Zhao, Fuxiang Zhou, Jian Zhou, Jing Jin, Jin Gu, Lin Shen

**Affiliations:** 1 Key Laboratory of Carcinogenesis and Translational Research (Ministry of Education), Peking University Cancer Hospital & Institute, No. 52, Fucheng Road, Haidian District, Beijing, 100142 China; 2 The Second Affiliated Hospital of Zhejiang University School of Medicine, No. 88, Jiefang Road, Hangzhou, Zhejiang China; 3 Peking University People’s Hospital, No. 11, Xizhimen Nandajie, Beijing, China; 4 Sun Yat-sen University Cancer Center, No. 651, Dongfeng East Road, Yuexiu District, Guangzhou, Guangdong China; 5 National Cancer Center/Cancer Hospital, Chinese Academy of Medical Sciences, Peking Union Medical College, No. 17, Panjiayuan Nanli, Chaoyang District, Beijing, China; 6 The Sixth Hospital Affiliated of Sun Yat-sen University, No. 19, Erheng Road, Yuancun, Tianhe District, Guangzhou, Guangdong China; 7 Tianjin Medical University Cancer Institute & Hospital, Huanhu West Road, Tiyuanbei, Hexi District, Tianjin, China; 8 Peking Union Medical College Hospital, No. 1, Shuaifuyuan, Dongcheng District, Beijing, China; 9 Chinese People’s Liberation Army General Hospital, No. 28, Fuxing Road, Haidian District, Beijing, China; 10 Shanxi Provincial Cancer Hospital, No. 3, Zhigong Xincun, Xinghualing District, Taiyuan, Shanxi China; 11 Huaxi Hospital of Sichuan University, No. 37, Guoxue Lane, Wuhou District, Chengdu, Sichuan China; 12 Nanfang Hospital of Southern Medical University, No. 1838, Guangzhou North Road, Guangzhou, Guangdong China; 13 Fudan University Shanghai Cancer Center, No. 270, Dongan Road, Xuhui District, Shanghai, China; 14 Fujian Medical University Union Hospital, No. 29, Xinquan Road, Gulou District, Fuzhou, Fujian China; 15 The First Affiliated Hospital of Zhengzhou University, No. 1, Jianhe East Road, Zhengzhou, Henan China; 16 The First Affiliated Hospital of Zhejiang University School of Medicine, No. 79, Qingchun Road, Zhejiang, Hangzhou China; 17 China-Japan Union Hospital of Jilin University, No. 126, Sendai Street, Changchun, Jilin, China; 18 Tongji University Shanghai East Hospital, No. 150, Jimo Road, Pudong New Area, Shanghai, China; 19 Jiangsu Provincial People’s Hospital, No. 300, Guangzhou Road, Nanjing, Jiangsu China; 20 The Seventh Affiliated Hospital of Sun Yat-sen University, No. 628, Zhenyuan Road, Shenzhen, Guangdong China; 21 Zhejiang University School of Medicine, No. 866, Yuhangtang Road, Zhejiang, Hangzhou China; 22 The First Affiliated Hospital of Xi’an Jiaotong University, No. 277, Yanta West Road, Xi’an, Shaanxi China; 23 Zhejiang Cancer Hospital, No. 38, Guangji Road, Banshanqiao, Gongshu District, Zhejiang, Hangzhou China; 24 Shandong Provincial Hospital, No. 324, Jingwuweiqi Road, Ji’nan, Shangdong China; 25 Zhongshan Hospital of Fudan University, No. 180, Fenglin Road, Xuhui District, Shanghai, China; 26 The First Hospital of China Medical University, No. 155, Nanjing North Street, Heping District, Shenyang, Liaoning China; 27 Sir Run Run Shaw Hospital of Zhejiang University School of Medicine, No. 3, Qingchun East Road, Zhejiang, Hangzhou China; 28 Xiangya Hospital of Central South University, No. 87, Xiangya Road, Changsha, Hunan China; 29 Xinqiao Hospital of Army Medical University, No. 83, Xinqiaozheng Street, Shapingba District, Chongqing, China; 30 The Fourth Affiliated Hospital of Harbin Medical University, No. 37, Yiyuan Street, Nangang District, Harbin, Heilongjiang China; 31 The First Affiliated Hospital of Soochow University, No. 188, Shizi Street, Canglang District, Suzhou, Jiangsu China; 32 The First Affiliated Hospital of Jilin University, No. 71, Xinmin Road, Changchun, Jilin, China; 33 The Third People’s Hospital of Shenzhen, No. 29, Bulan Road, Longgang District, Shenzhen, Guangdong China; 34 Peking University Shenzhen Hospital, No. 1120, Lianhua Road, Futian District, Shenzhen, Guangdong China; 35 Shandong Qianfoshan Hospital, No. 16766, Jingshi Road, Lixia District, Ji’nan, Shandong China; 36 Zhongnan Hospital of Wuhan University, No. 169, Donghu Road, Wuchang District, Wuhan, Hubei China; 37 307 Hospital of the Chinese People’s Liberation Army, Road 8, Dong Street, Fengtai Distinct, Beijing, China; 38 Affiliated Hospital of Shandong Academy of Medical Sciences, No. 38, Wuyingshan Road, Tianqiao District, Ji’nan, Shandong China; 39 Beijing Friendship Hospital, No. 95, Yong’an Road, Xicheng District, Beijing, China; 40 Shandong Cancer Hospital, No. 440, Jiyan Road, Ji’nan, Shandong China; 41 The Fourth Hospital of Hebei Medical University, No. 12, Jiankang Road, Shijiazhuang, Hebei China; 42 Liaoning Cancer Hospital & Institute, No. 44, Xiaoheyan Road, Dadong District, Shenyang, Liaoning China; 43 Ruijin Hospital of Shanghai Jiaotong University School of Medicine, No. 197, Ruijin 2nd Road, Shanghai, China; 44 Changhai Hospital, No. 168, Changhai Road, Yangpu District, Shanghai, China; 45 Harbin Medical University Cancer Hospital, No. 150, Haping Road, Nangang District, Harbin, Heilongjiang China

**Keywords:** Consensus, Colorectal cancer, Lung metastases, China, Multidisciplinary therapy

## Abstract

The lungs are the second most common site of metastasis for colorectal cancer (CRC) after the liver. Rectal cancer is associated with a higher incidence of lung metastases compared to colon cancer. In China, the proportion of rectal cancer cases is around 50%, much higher than that in Western countries (nearly 30%). However, there is no available consensus or guideline focusing on CRC with lung metastases. We conducted an extensive discussion and reached a consensus of management for lung metastases in CRC based on current research reports and the experts’ clinical experiences and knowledge. This consensus provided detailed approaches of diagnosis and differential diagnosis and provided general guidelines for multidisciplinary therapy (MDT) of lung metastases. We also focused on recommendations of MDT management of synchronous lung metastases and initial metachronous lung metastases. This consensus might improve clinical practice of CRC with lung metastases in China and will encourage oncologists to conduct more clinical trials to obtain high-level evidences about managing lung metastases.

**Electronic supplementary material:**

The online version of this article (10.1186/s13045-019-0702-0) contains supplementary material, which is available to authorized users.

## Overview and epidemiology of lung metastases in colorectal cancer

The incidence of colorectal cancer (CRC) continues to increase each year. In 2015, the incidence and mortality of CRC is ranked fifth among all malignant tumors in China [1]. In recent years, the widespread use of chest CT scans has resulted in a continuous increase in the number of CRC patients who are diagnosed with lung metastases. Retrospective data from 1996 to 2017 from Peking University Cancer Hospital show that lung metastases accounted for 32.9% of all metastatic CRCs (mCRC) and the tumors in 24.5% of mCRC patients first metastasized to the lung [2]. Currently, the lungs are the second most common site of metastasis for CRC after the liver. As patients with rectal cancer are prone to lung metastases [3, 4] and the proportion of rectal cancer cases in China (nearly 50%) is significantly higher than in Western countries (around 30%) [5–8], the diagnosis and treatment of lung metastases in CRC are more significant clinical problems in China.

Compared to other distal metastases, lung metastases have relatively slower growth and better overall survival [9]. Therefore, the treatment strategy for lung metastases cannot be made completely according to that for metastases at other sites (e.g., liver, peritoneum). However, there are no guidelines or expert consensus for lung metastases in CRC. Therefore, the Chinese College of Surgeons Expert Committee on Multidisciplinary Therapy and The Committee of Colorectal Cancer of the China Anti-Cancer Association have conducted an extensive discussion and reached a consensus for the recommendation of the multidisciplinary management for lung metastases in CRC.

Lung metastases can be classified into synchronous metastases and metachronous metastases based on the interval between the primary tumor and lung metastases appear. This consensus considers the perspective of clinical operability and refers to the latest treatment mode for liver metastases [10] to define synchronous lung metastases as “lung metastases that are discovered during the diagnostic workup for the primary tumor of CRC,” whereas metachronous lung metastases are defined as “lung metastases that are found after diagnostic examinations.”

Lung metastases are classified as initial metastases and non-initial metastases according to the sequence of lung metastases and other distal metastases. Initial lung metastasis is defined as lungs being the site of the first distal metastases, regardless of whether it is accompanied by other distal metastases. This includes all synchronous lung metastases and initial metachronous lung metastases (lung metastases that appear during preoperative neoadjuvant therapy or after resection of the primary lesion) and accounts for 74.4% of all lung metastases [2]. These metastases are the primary focus of discussion in this consensus. In contrast, non-initial lung metastases are developed during treatment for other metastatic diseases so they are all metachronous metastases.

Lung metastases can be classified as isolated lung metastases and non-isolated lung metastases depending on whether it is accompanied by extrapulmonary metastases (see Additional file 1).

Among patients with initial lung metastases, 37.7–44.5% have isolated lung metastases, of which only 21.1–32.5% of patients can undergo radical surgery for lung metastases [2, 11]. The remaining patients with isolated lung metastases have no opportunity to undergo radical treatment. For this population, there is a great uncertainty on the selection of systemic therapy, whether other local treatments of lung metastases can be conducted, and how to manage the primary CRC lesions. Among patients with non-isolated lung metastases, 38.6–55.5% have comorbid liver metastases [2, 11], of which most patients cannot receive radical treatment of all metastatic lesions. Is there survival benefit if local treatment is conducted on the primary lesions and/or liver metastases? This consensus provides recommendations for clinical diagnosis and treatment decisions for the aforementioned problems. In addition, this consensus also discusses some specific problems related to non-initial lung metastases such as the management of lung metastases after resection of liver metastases.

## Diagnosis and differential diagnosis of lung metastases in CRC

### Imaging diagnosis

Unless lymphangitic carcinomatosis or extensive pleural metastases occur, CRC patients with lung metastases usually do not show respiratory signs or symptoms. Therefore, it is recommended that high-resolution chest CT scans be used, whereas other imaging methods such as chest X-rays and MRI are not recommended. It is recommended that enhanced chest CT scans be used for the diagnosis of mediastinal or hilar lymph node metastases.

#### Risk factors supporting lung metastases diagnosis

The risk factors supporting lung metastases diagnosis are age of onset > 70 years, multiple nodules in both lungs, metachronous intrapulmonary nodules, pleural thickening or effusion, rectal cancer (particularly middle or lower rectal cancer), locally advanced CRC (particularly extramural vascular invasion), higher N stages, lymphovascular invasion at the primary lesion, elevated preoperative CEA levels, *KRAS* mutations in the primary lesion, and presence of liver metastases or other extrapulmonary metastases [3, 4, 12–20].

#### Lymphangitic carcinomatosis

The signs of lymphangitic carcinomatosis are irregular or nodular thickening of peripheral vascular and tracheal bundles, interlobular septa uniform or nodular thickening with normal lobular morphology or angular changes, and regional lymph node enlargement. Other signs include diffuse intrapulmonary nodules, pleural hypertrophy, and pleural effusion.

#### Differential diagnosis

Lung metastases in CRC should be differentiated from other malignant nodules such as primary lung cancer and benign diseases including benign non-specific nodules, infectious lesions, and immune disorders.

### Pathological diagnosis

In pathology, CRC lung metastases appear as moderately to highly differentiated adenocarcinomas, with relatively large glandular lumen and tall epithelial cells. Primary lung adenocarcinomas mostly exhibit an acinar growth pattern with relatively small glandular lumen. The epithelial cells often appear as hobnail-like cells. Adenocarcinomas in situ showing lepidic growth are often seen nearby. For poorly differentiated metastatic colorectal adenocarcinomas and unique types of adenocarcinomas such as mucinous adenocarcinoma, signet ring cell carcinoma, and poorly differentiated primary lung adenocarcinomas, it is difficult to differentiate them by morphology, thus immunohistochemistry and detailed medical history should be used for differentiation. CRC lung metastases commonly stain positive for CK20, CDX-2, and SATB2, whereas primary lung adenocarcinomas commonly stain positive for CK7, TIF-1, and napsin A [21]. Other neoplasms should be considered in the differentiation as well (e.g., neuroendocrine tumor, pneumocytoma, adenocystic carcinoma, myxoepithelioma).

In addition, the consensus recommends routine testing for *KRAS*, *NRAS*, and *BRAF* mutation, as well as status of microsatellite instability (MSI) or function of mismatch repair (MMR) proteins [22]. Because *KRAS* mutation is involved in lung metastasis, the frequency of *KRAS* mutation in patients with lung metastases is high [13]. It is also recommended that HER2 immunohistochemical detection be routinely conducted.

Patients with lung metastases exhibit some molecular characteristics different from the primary tumors; therefore genetic analysis of lung metastasis should be conducted when possible to better aid the oncologist in determining a treatment regimen. For patients whose tissue specimens from metastatic lesions cannot be obtained, liquid biopsy can be considered for testing of relevant genes and molecular markers [23]. However, this strategy has not been validated by large-scale clinical studies, and the interpretation of results remains debatable.

## General guidelines for multidisciplinary therapy of lung metastases in CRC

**Fig. 1:**
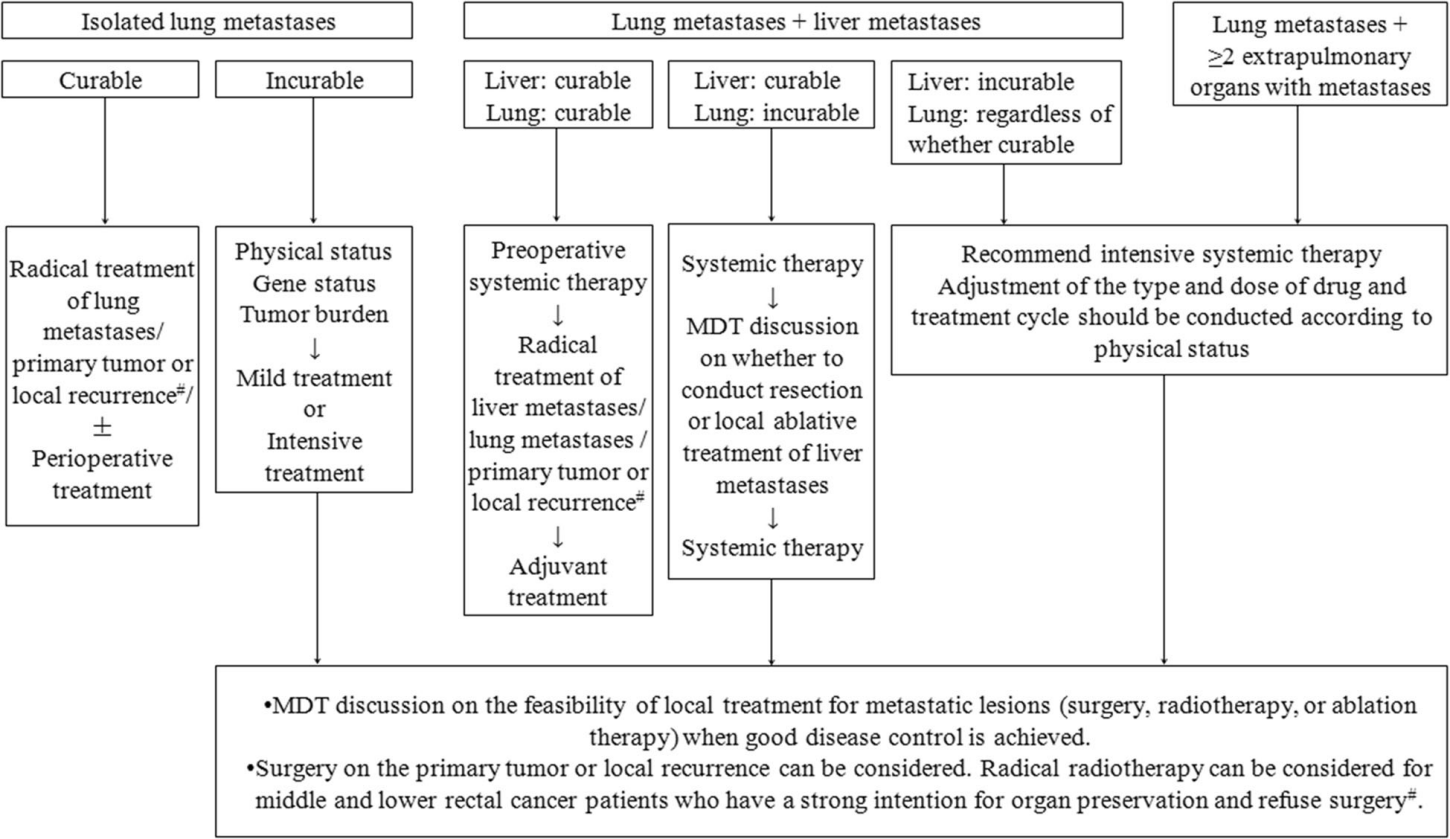
Principles of treatment for lung metastases. Note 1: Number sign is patients with primary tumor or local recurrence. If the primary tumor or local recurrence that cannot be radically treated initially, reassess the possibility of radical treatment after (intensive) systemic therapy: (1) if the lesion has converted to a curable tumor, then the above process can be used as a reference; (2) if the tumor is still incurable, a comprehensive therapy protocol should be formulated after multidisciplinary discussion (in such cases, the lesion could be managed as “an incurable metastatic lesion”). Note 2: For lung metastases + extrapulmonary metastases at any site other than the liver, please refer to the treatment principles for “lung metastases + liver metastases.” Note 3: Non-initial lung metastases are a highly heterogenous group of diseases. In contrast to initial lung metastases, these patients have previously received drug treatment, and further drug efficacy is relatively low. For these patients, it is recommended that a final decision be made after the MDT team has comprehensively examined the patient’s physical status, efficacy and adverse events from previous treatment, drug discontinuation duration, and the biological behavior of tumors

Overall, for patients with curable lung metastases, the treatment goal is to achieve the status of no evidence of disease (NED) and reduce recurrence risk, and for those with incurable disease even after intensive systemic therapy, the goal is to prolong survival duration and improve life quality. The general principle of the management of lung metastases is that comprehensive therapy should be conducted after multidisciplinary discussion, because the number, site, size, and genotype of lung metastases, primary tumor location, and extrapulmonary metastases affect prognosis and treatment decisions. Therapeutic approaches include systemic therapy, radical local treatment (e.g., R0 surgical resection, stereotactic radiation therapy, and ablation therapy), and local palliative treatment. Multidisciplinary discussion should combine the patient’s clinical characteristics with accessibility to medical resources to determine treatment goals and thereby formulate a rational and orderly comprehensive treatment strategy (Fig. 1).

During the early phase of treatment, the multidisciplinary therapy (MDT) team should determine whether the metastatic lesions and the primary tumor are curable. Usually, systemic therapy is first administered to determine treatment responses and the biological behavior of the tumors before radical treatment on all lesions in patients who can technically achieve NED. For patients who cannot achieve NED, it is recommended that a decision be made on whether management of local lesions (including primary tumor or local recurrence) should be conducted under the guidance of the MDT team when good systemic disease control is achieved (Fig. 1). During treatment, the biological behavior of tumors, treatment responses, and extrapulmonary metastases should be monitored and timely adjustment to treatment expectations and regimen be conducted.

Even though lung metastases develop slowly and are usually not the primary factors affecting survival and prognosis, the MDT team should be alerted of the possibility of lymphangitic carcinomatosis. This type of intrapulmonary metastases has extremely poor outcomes, and local treatment is usually not recommended for this population of patients. Systemic therapy is the mainstay treatment, and the drugs used should be determined according to the patient’s medical history, physical status, and genetic status so that the most effective drug regimens can be selected. In addition, if emergencies such as bleeding, perforation, and obstruction in the primary tumor or local recurrent lesion occur during diagnosis and treatment, then it is recommended that these emergency complications be managed (e.g., surgical resection, stent implantation, or colostomy) before the patients are treated according to the management procedure for asymptomatic patients. The subsequent discussions will focus on patients with no emergency complications relating to primary/local recurrent lesions.

High-level clinical evidence is limited; therefore, this consensus encourages Chinese oncologists to conduct multicenter clinical studies and encourages individual centers to participate in these future multicenter trials. This consensus also encourages individual cancer centers to include every patient with oligometastatic disease and MDT in a prospectively created registry for quality control and future outcome analysis.

## Management of synchronous lung metastases

### Management of isolated and resectable lung metastases

Around 9.4–12.2% of patients with lung metastases are suitable for local radical treatment [2, 11], which includes R0 surgery, radiotherapy, and ablation therapy. Even though there are no mature randomized controlled trials, surgery is regarded as the local treatment method with the clearest benefit. Most retrospective studies have found that surgery is superior to chemotherapy alone. After resection of the intrapulmonary lesions, the 5-year survival rate is 35–70% and the 10-year survival rate is 20–30% [24–42] but the 5-year survival rate for patients who received chemotherapy alone is only around 20% [43]. Therefore, it is recommended that aggressive surgical resection be conducted on patients with resectable lung metastases. Radiotherapy and ablation therapy can be considered as alternative measures for patients who are not suitable for surgery due to tumor site, expected residual lung function, patient tolerance, or patient willingness [44].

#### Principles of surgical resection

Generally, the first choice for resection of lung metastases is sublobar resection such as wedge resection or segmental resection of the lung. However, under some rare circumstances, pulmonary lobectomy can be conducted as a last resort when the tumor is deep or when there is massive intraoperative bleeding. Patients who receive pulmonary lobectomy have relatively poor prognosis. Even if R0 surgery is conducted, patients with the following factors will have poor prognosis: multiple lung metastases, hilar or mediastinal lymph node metastases, preoperative CEA elevations, large metastatic lesions, shorter disease-free interval (DFI), older age, advanced primary tumor stage, rectal primary tumor, and non-R0 surgery (Table 1).

**Table 1:** Prognostic factors after resection of colorectal cancer lung metastases

Factors resulting in poor prognosis	Description
Multiple metastatic lesions	Poor survival if > 1 lesion [24, 26, 34, 35, 37]
	Poorer survival if the number of metastasis > 4 or metastases are present in both lungs [28, 29]
Hilar/mediastinal lymph node metastases present	Poor survival if lymph node metastases are present [30, 32, 33, 35, 37, 39, 41]
High preoperative CEA levels	Poor survival if > 5 ng/ml [33–35, 37, 40, 41]
Large tumor diameter	The larger the tumor, the poorer the survival [34]
Short disease-free interval (DFI)	Poor survival if DFI < 24 months [26]
Older age	Poor survival if > 70 years [29]
Advanced primary tumor stage	Advanced stage of primary tumor results in poor prognosis [36]
Primary tumor located at the rectum	Rectal cancer has a poorer survival than colon cancer [25]
R1 or R2 resection	R1 or R2 resection is associated with poor survival [33, 42]
Pulmonary lobectomy	Pulmonary lobectomy has poorer survival than wedge resection or segmental resection of the lung [40]

For patients with no suspected hilar/mediastinal lymph node metastasis in preoperative examinations, routine lymph node dissection during surgery is not required. If lymph node metastases are suspected, then lymph node biopsy or dissection can be considered during surgery.

#### Perioperative treatment

For resectable lung metastases, the goal of perioperative treatment is to increase the R0 resection rate and decrease the risk of postoperative recurrence. Preoperative systemic therapy can also aid in determining the biological behavior of the tumors. However, there is currently insufficient clinical trial data on preoperative drug treatment in CRC lung metastases, so it is not clear whether this will improve the disease-free survival (DFS) and overall survival (OS) of patients.

For rectal cancer, no studies on perioperative treatment in synchronous lung metastases have been performed to date. It is recommended that sufficient systemic therapy be conducted before surgery for primary tumors and lung metastases and preoperative neoadjuvant rectal radiotherapy be conducted for patients with T3–4 or N+ middle or lower rectal cancer. After surgery, the entire perioperative treatment duration of less than 6 months can be completed according to the tumor response of preoperative treatment and the patient’s physical condition. For rectal cancer patients who did not undergo preoperative treatment, it is recommended that up to 6 months of combined oxaliplatin-based chemotherapy ± radiotherapy be conducted after surgery according to extrapolation of results from the IDEA study (only colon cancer involved) and the SCOT study (both colon cancer and rectal cancer involved) [45, 46]. The specific implementation protocol should be performed under the guidance of an MDT team (Table 2).

**Table 2:** Treatment strategy for patients who initially have resectable lung metastases

Clinical situation	Alternative strategies
Middle and lower rectal cancer: T3–4 or N+	1. Preoperative systemic drug treatment/rectal neoadjuvant radiotherapy, resection of primary lesion/lung metastases, adjuvant chemotherapy
	2. Preoperative systemic drug treatment/rectal neoadjuvant radiotherapy, resection of primary lesion, systemic drug treatment, resection of lung metastases, adjuvant chemotherapy
Middle and lower rectal cancer: T1-2N0; Upper rectal cancer and colon cancer: T1–4N0–2	1. Preoperative systemic drug, resection of primary lesion, resection of lung metastases, adjuvant chemotherapy
	2. Resection of primary lesion, systemic drug treatment, resection of lung metastases, adjuvant chemotherapy
	3. Resection of primary lesion, resection of lung metastases, adjuvant chemotherapy

Non-surgical local treatment measures can be considered for resectable lung metastases in patients who are not suitable for or refuse surgery. These measures mainly include stereotactic body radiation therapy (SBRT) and ablation therapy. Similar to the principles of surgical treatment, the status of the primary lesion must be assessed before SBRT or ablation therapy and these treatments should be conducted under conditions in which there is good overall tumor control. The principles of systemic therapy are similar to those of perioperative treatment. For single lung metastases, radiofrequency ablation should be first considered when the lesion is located at the outer zone. If the lesion is located at the middle zone, then both radiofrequency ablation and radiotherapy can be considered; and if the lesion is located in the inner zone or near blood vessels, then radiotherapy should be considered first [44]. For multiple lung metastases, a corresponding treatment method can be decided after multidisciplinary discussion.

### Management of isolated and potentially resectable lung metastases

Currently, there is no clear definition of “potentially resectable lung metastases.” In most patients who initially have unresectable lung metastases, the reasons for unresectability are widespread distribution of lung metastases and a large number of lung metastases. However, in a minority of patients, the reason is the location or size of the metastatic lesions, or that the metastatic lesions are close to the hilus of the lung or important blood vessels or organs. These patients may achieve the opportunity to undergo R0 surgery through intensive conversion therapy. A small-sample study showed that the conversion rate of lung metastases is 5.7–7.1% [47, 48] (Table 3).

**Table 3:** Clinical strategy for potentially resectable lung metastases

Clinical situation	Alternative strategies
Patients who have initial potentially resectable lung metastases^1^	1. Conversion therapy, resection of primary tumor, resection of lung metastases, postoperative adjuvant therapy
	2. Resection of primary tumor, conversion therapy, resection of lung metastases, postoperative adjuvant chemotherapy^2^

^1^MDT team discussion is needed to determine if the lesion is potentially resectable

^2^After successful conversion and R0 resection of initial potentially resectable lung metastases, it is recommended that the preoperative treatment regimen be used as postoperative therapy

### Management of isolated and unresectable lung metastases

Palliative treatment should be conducted on patients with unresectable lung metastases and includes systemic therapy and palliative local treatment. Treatment goals and drug toxicity should be fully considered for systemic therapy [2]. Combination chemotherapy or monochemotherapy ± targeted therapy can be considered. Regular follow-up can be considered for asymptomatic patients with isolated lung metastases, particularly patients with small metastases (e.g., lesions < 1 cm) and good prognosis. Regorafenib may be a choice after failure with first-line and second-line treatment [49]. Please refer to the 2017 National Health and Family Planning Commission *Chinese Protocol of Diagnosis and Treatment of Colorectal Cancer* for specific drug options [22]. As some patients with lung metastases may have a long survival duration, the gene status of the metastatic lesions might change and thus influence further systemic therapy outcomes after undergoing multiple lines of treatment or a long treatment duration. When possible, re-biopsy of the intrapulmonary lesions or liquid biopsy can be conducted when disease progression occurs to fully understand gene status in the tumor [23].

### Management of lung metastases with extrapulmonary metastases at any other sites

Lung metastasis is the subtype with the best prognosis among metastasis types [9]. Therefore, when lung metastases are accompanied by distal metastases at any other site, these other distal metastases are usually the main consideration for treatment, except when the burden of lung metastases is large and the patient is symptomatic. Thus, this consensus uses liver metastases as an example for corresponding recommendations (Table 4). Treatments for metastases at other sites should refer to the treatments outlined for liver metastases here. For potentially resectable or unresectable metastatic lesions, if these lesions can be converted to resectable by treatment, then they should be included in corresponding routes for management.

**Table 4:** Treatment strategy for lung metastases with liver metastasis only

Clinical situation		Alternative strategies
Lung metastases	Liver metastases	
Curable	Curable	Radical local treatment of the primary tumor, lung metastases, and liver metastases^1^ in stages. Administer 6 months of perioperative treatment before and after local treatment^2^
Curable	Incurable	Systemic treatment^3^
Incurable	Curable	Elective radical local treatment^4^ for liver metastases can be conducted on the basis of effective systemic treatment^3^
Incurable	Incurable	Systemic treatment^3^

^1^It is recommended that the local treatment sequence and method be decided after MDT team discussion. It is currently believed that the type of resection for lung metastases has mild effects on the patient prognosis. If both liver metastases and lung metastases are technically resectable lesions, simultaneous or sequential resection of the lesions can be conducted. It is recommended that metastatic lesions with the highest difficulty be resected first. However, considering that reduced pulmonary function after resection of lung metastasis may affect surgical anesthesia, it is usually recommended that liver metastases be resected first when there are no differences of surgical difficulties between lung metastases and liver metastases [55]. SBRT or radiofrequency ablation is also effective for local treatment of lung metastases. This is particularly so for non-technically unresectable lung metastases, in which the use of SBRT or ablation therapy is highly recommended [56–59]

^2^Perioperative treatment includes neoadjuvant therapy and adjuvant therapy. Neoadjuvant chemotherapy can reduce the preoperative tumor volume and reduce the formation of micrometastases and increase radical resection rate of surgery. In order to limit the occurrence of drug-induced liver injury, the duration of neoadjuvant chemotherapy is usually limited to 2–3 months. In addition, the patient’s physical condition, *RAS* and *BRAF* gene status, and tumor burden should be considered to decide whether chemotherapy should be combined with targeted therapy

^3^It is necessary to consider the patient’s physical status, location of primary tumor, molecular biology characteristics, and prognostic status before determining the systemic treatment regimen

^4^For patients with lung metastases that are unable to undergo technical radical resection, resection of liver metastases may provide survival benefits [60]. However, the selection of these patients should be based on MDT team discussion

If there are ≥ 2 other metastasis sites in addition to lung metastases, then this condition is no longer considered to be oligometastases. This consensus recommends systemic palliative therapy be the main approach. During treatment, MDT team discussions should be conducted to determine whether to use local treatment according to drug efficacy and the biological behavior of the tumors (Table 5).

**Table 5:** Treatment strategy for lung metastases with two and above extrapulmonary metastasis sites

Clinical situation		Alternative strategies
Lung metastases	Extrapulmonary metastases	
Curable	Curable	Mainly systemic therapy^1^. This suitable patients for surgical resection of lung metastases and other metastatic lesions should be carefully selected^2^
Curable	Incurable	Systemic therapy^1^
Incurable	Curable	Systemic therapy^1^
Incurable	Incurable	Systemic therapy^1^

^1^It is necessary to consider the patient’s physical status, location of primary tumor, molecular biology characteristics, and prognostic status before determining the systemic therapy regimen

^2^In addition to liver metastases, extrapulmonary metastases may be peritoneal metastases, pelvic implantation metastases, ovarian metastases, abdominal lymph node and superficial lymph node metastases, brain metastases, and bone metastases. Currently, there is still a debate on whether resection of the aforementioned extrapulmonary metastases can result in survival benefits for patients. Only a small number of studies found that R0 resection of some organ metastases (such as peritoneal metastases, ovarian metastases, and abdominal metastases) may improve patient survival. Therefore, caution must be exercised when selecting patients for local treatment of lung metastases and other metastatic lesions. It is recommended that systematic therapy be conducted first [44]

Resectability assessment of multi-organ metastatic lesions (including lung metastases), selection of surgery timing, and comprehensive therapy require MDT team discussion before determining the treatment strategy. With regard to the colorectal primary tumor and multi-organ metastases, the assessment method is listed in Table 6.

**Table 6:** Assessment methods of the colorectal primary tumor and multi-organ metastases

Tumor lesion	Assessment methods
Primary tumor	1. Colonoscopy should be conducted to determine the location of the primary tumor, tumor size, the proportion of the intestinal lumen that the tumor occupies, and biopsy should be used to confirm the pathology of the tumor (including molecular biology tests, *RAS/BRAF* gene status, microsatellite instability status, and HER2 status)
	2. Enhanced CT of the entire abdomen
Lung metastases	1. High-resolution chest CT scan^1^
	2. Enhanced chest CT scan (when mediastinal lymph node metastases are present)
	3. Evaluation of lung function
Liver metastases	Contrast-enhanced MRI of the liver^1^
Other metastases	1. Contrast-enhanced MRI of the pelvis (when pelvic implantation metastases are present)
	2. Bone ECT (when symptoms associated with bone metastases are present)
	3. PET-CT^2^

^1^For patients with only liver metastases as extrapulmonary metastases, it is recommended that high-resolution chest CT scan be used for resectability assessment of lung lesions. Additionally, enhanced CT of the entire abdomen and contrast-enhanced MRI of the liver should be used to for resectability assessment of liver metastases [61–63]. In addition to assessment for technical resectability, biological behavior assessment should also be considered. Currently, it is believed that patients with more than five liver metastases, more than three lung metastases, and who exhibit progression after neoadjuvant treatment have poor outcomes after radical resection [64, 65]

^2^PET-CT is only used when it is impossible to determine the nature of lung lesions and extrapulmonary lesions. Currently, it is believed that PET-CT has greater value for intrapulmonary lesions > 1 cm [66]

Currently, it is believed that chemotherapy combined with targeted therapy cannot convert initially unresectable multi-organ metastases (including lung metastases) to resectable multi-organ metastases. However, a minority of patients who are particularly sensitive to chemotherapy or chemotherapy combined with targeted therapy may undergo successful conversion.

## Management of special situations in rectal cancer with synchronous lung metastases

After locally advanced (T3–4 or N+) middle or lower (≤ 10 cm from the anal margin) rectal cancer has been treated with neoadjuvant therapy, particularly total neoadjuvant therapy (TNT), good long-term control can be achieved for the primary tumor, with a pathological complete response (pCR) of 25–30%. Even though there is a lack of data from large-sample clinical studies for neoadjuvant radiochemotherapy efficacy and strategy in metastatic middle and lower rectal cancer, surgery of middle and lower rectal cancer may significantly affect quality of life (e.g., postoperative complication, stoma care). Therefore, with regard to locally resectable middle and lower rectal cancer with synchronous lung metastases, the following recommendations are made for the management of the primary tumor: When the treatment goal is NED, it is recommended that preoperative systemic therapy and neoadjuvant radiotherapy for the primary tumor be conducted before radical resection of the primary tumor and metastatic lesions. The management order for the primary tumor and metastatic lesions requires MDT team discussion. If the patient has a strong intention for organ preservation and refuses surgery, radical resection of the metastatic lesions can be conducted after TNT treatment and the status of the primary tumor should be closely monitored during follow-up.

## Management of initial metachronous lung metastases

Initial metachronous lung metastases mainly include lung metastases that appear during the planned neoadjuvant treatment period or after resection of the primary tumor. Please refer to the corresponding section in synchronous lung metastases for the former situation. With regard to the latter situation, if this is accompanied by local recurrence and the local recurrent lesion is resectable, then the local recurrence lesion should be considered as a “primary lesion” and management should be conducted according to “synchronous lung metastases.” If the local recurrent lesion is unresectable, then this should be considered as an unresectable metastatic lesion and management should be conducted according to the route in Fig. 1. The overall treatment principles are similar to synchronous lung metastases, which will not be discussed here. In this section, we will only discuss perioperative treatment for isolated resectable lung metastases.

This consensus recommends that patients who meet the criteria for resection undergo surgery as the initial treatment [11, 50, 51]. Because perioperative therapy may be superior to surgery alone, postoperative chemotherapy is reasonable and the regimens should be made based on treatments for locally advanced CRC, with oxaliplatin-based treatments being recommended. If preoperative neoadjuvant therapy is an irinotecan-based chemotherapy regimen, then the original regimen can be continued as postoperative adjuvant therapy in patients in which the treatment is effective. The total duration of perioperative chemotherapy should not exceed 6 months [19, 31, 50, 52–54].

## Follow-up

Regular follow-up is recommended after NED achieved with local ablative treatment in CRC with lung metastases. Plain or enhanced chest CT scan should be conducted once every 6 months for 3 years, and then once a year, for a total of 5 years. Other post-treatment surveillance is referred to *Chinese Protocol of Diagnosis and Treatment of Colorectal Cancer* [22].

## Conclusion and perspective

In conclusion, this consensus is based on Chinese oncologists’ opinions and limited clinical evidence. We recognized the urgency to conduct randomized prospective controlled clinical studies in the management of CRC lung metastasis. In summary, we hope this consensus provides guidance in treating CRC lung metastasis and encourage in this field.

## Additional file

Additional file 1: Classification of CRC lung metastases. (DOC 33 kb)

## Abbreviations

CRC: Colorectal cancer
DFI: Disease-free interval
DFS: Disease-free survival
mCRC: Metastatic colorectal cancer
MDT: Multidisciplinary therapy
MMR: Mismatch repair
MSI: Microsatellite instability
NED: No evidence of disease
OS: Overall survival
pCR: Pathological complete response
SBRT: Stereotactic body radiation therapy
TNT: Total neoadjuvant therapy
