# Supplementary material for: Expert consensus on multidisciplinary therapy of colorectal cancer with lung metastases (2019 edition)
Source: J Hematol Oncol. 2019 Feb 14;12:16. doi: 10.1186/s13045-019-0702-0 (PMC6376656; doi:10.1186/s13045-019-0702-0)
Supplement: Supplementary file 1 — Classification of CRC lung metastases. (DOC 33 kb) [file 13045_2019_702_MOESM1_ESM.doc]

**Additional File 1.** **Classification of CRC Lung Metastases**

***According to the time when the primary tumor and lung metastases appear:***

Synchronous lung metastases: Lung metastases found during initial staging of CRC.

Metachronous lung metastases: Lung metastases found after baseline examinations.

***According to whether there are accompanying extrapulmonary metastases:***

Isolated lung metastases: No accompanying extrapulmonary metastases (regardless of whether the primary tumor or local recurrence lesion is present).

Non-isolated lung metastases: Accompanied by extrapulmonary metastases (regardless of whether the primary tumor or local recurrence lesion is present).

***According to the sequence of appearance of lung metastases and other distal metastases:***

Initial lung metastases: Lung being the site of the first distal metastases, regardless of whether it is accompanied by other distal metastases. This includes all synchronous lung metastases and initial metachronous lung metastases (lung metastases that appear during preoperative neoadjuvant therapy or after resection of the primary lesion).

Non-initial lung metastases: Lung metastases that appear during treatment for other metastatic diseases.

Table S1. Classification of lung metastases

| Clinical situation | Classification of lung metastases | | | | |
| --- | --- | --- | --- | --- | --- |
| Whether discovered during cancer staging | Synchronous | | Metachronous | | |
| Whether there are extrapulmonary metastases | Isolated | Non-isolated | Isolated | Non-isolated | |
| Whether lung metastasis is the first metastasis | Initial | | | | Non-initial |
